# Microbial signatures in oral sites of patients with primary Sjögren’s syndrome: Association with salivary gland hypofunction

**DOI:** 10.71150/jm.2501030

**Published:** 2025-06-30

**Authors:** Sarah Kamounah, Arjun Sarathi, Christiane Elisabeth Sørensen, Manimozhiyan Arumugam, Anne Marie Lynge Pedersen

**Affiliations:** 1 Section for Oral Biology and Immunopathology/Oral Medicine & Pathology, Department of Odontology, Faculty of Health and Medical Sciences, University of Copenhagen, Nørre Allé 20, 2200 Copenhagen N, Denmark; 2 Novo Nordisk Foundation Center for Basic Metabolic Research, Faculty of Health and Medical Sciences, University of Copenhagen, Blegdamsvej 3B, 2200 Copenhagen N, Denmark; 3 Section for Clinical Oral Microbiology, Department of Odontology, Faculty of Health and Medical Sciences, University of Copenhagen, Nørre Allé 20, 2200 Copenhagen N, Denmark

**Keywords:** primary Sjögren’s syndrome, oral microbiome, hyposalivation

## Abstract

This study aimed to determine if the microbiota in four different oral sites and the oral health status differ between patients with primary Sjögren’s syndrome (pSS), non-pSS sicca symptoms, and healthy controls. All participants underwent an interview and clinical oral examination. Stimulated whole saliva (SWS), supragingival plaque (SGP), buccal mucosa tissue (BLM), and tongue scrape (TGS) samples from 23 pSS patients, 36 patients with sicca symptoms, not fulfilling the classification criteria for pSS (non-pSS sicca), and 21 age-matched healthy controls (HC) were analyzed using V3–V4 16S rRNA gene amplicon sequencing, and determination of amplicon sequence variants (ASVs). PSS and non-pSS sicca patients did not differ with respect to oral health status, saliva flow rates, abundance of predominant genera, relative abundance on genus level or bacterial diversity in any of the oral sites. Both patient groups differed significantly from the healthy control group in the abundance of 61 ASVs across all sites. The alpha-diversity was lower in SGP from non-pSS sicca patients (p = 0.019), and in TGS from pSS patients (p = 0.04). The proportion of variation in the beta-diversity across all four sites could be explained by the diagnosis (pSS, non-pSS sicca, and HC). However, subgrouping of patients according to their stimulated salivary flow rates (SWS > 0.7 ml/min versus SWS ≤ 0.7 ml/min), revealed significantly different abundance of three ASVs in SWS, 11 in SGP, and six in TGS. Our findings suggest that hyposalivation rather than pSS itself modifies the microbial composition in oral site-specific patterns leading to oral diseases.

## Introduction

Primary Sjögren’s syndrome (pSS) is a chronic autoimmune disease characterized by focal lymphocytic infiltration of the exocrine glands, predominantly the salivary and lacrimal glands, resulting in glandular dysfunction and severe oral and ocular dryness. A large variety of extraglandular manifestations including fatigue, myalgia, arthralgia, central and sensory peripheral neuropathy may also be observed (Brito-Zerón et al., 2016). The processes underlying the humoral and cellular autoimmune reactions observed in pSS, remain unclear. The etiology is unknown but considered multifactorial, involving an interplay of genetic predisposition, environmental factors, and epigenetic factors (Mariette and Criswell, 2018; Thorlacius et al., 2023). Among patients, the female-to-male ratio is 9:1, the incidence peaks around 40–50 years of age, and the global estimated prevalence ranges between 0.01% and 0.72% (Brito-Zerón et al., 2016).

Saliva is essential to maintain oral health. It provides antimicrobial activity via a large variety of proteins and peptides, neutralizes acids from food and bacteria via salivary buffering systems, contributes to the formation of the dental and mucosal pellicle, dilutes food detritus and bacteria, and it mechanically cleanses the oral cavity, and constantly covers and lubricates the oral mucosa, thereby enhancing the mucosal barrier function and a balanced oral microbiota (Dawes et al., 2015; Lynge Pedersen and Belstrøm, 2019). Salivary gland hypofunction can lead to oral microbial dysbiosis due to impaired oral clearance, impaired salivary buffer capacity and altered salivary composition, and consequently an increased risk of developing oral diseases. Thus, patients with pSS have a higher risk of developing dental caries and oral candidiasis (Pedersen et al., 2005; Yan et al., 2011).

During the last decade, emerging evidence suggests that imbalances in the complex interaction between the microbiota, particularly the gut microbiome, and host immune system, resulting in dysbiosis and deregulation of the immune response, contribute to the pathogenesis of a multitude of chronic immune-mediated diseases (Fujiwara et al., 2020). It has been demonstrated that patients with Sjögren’s syndrome (SS) present gut microbial dysbiosis, which is also related to disease severity (Mandl et al., 2017; Mendez et al., 2020; Moon et al., 2020). In addition, rheumatic arthritis, systemic sclerosis, and lupus erythematosus display gut dysbiosis (Hevia et al., 2014; Nguyen et al., 2023; Scher et al., 2013; Xu et al., 2019; Yu et al., 2022; Zhang et al., 2015). While the role of the oral microbiome in the pathogenesis of pSS remains elusive, the number of studies on the oral microbiome in patients with pSS has increased in recent years. The microbiome has been analyzed in different oral sites and samples, including saliva (Rusthen et al., 2019; Sembler-Møller et al., 2019; Sharma et al., 2020; Siddiqui et al., 2016; Tseng et al., 2021; Zhou et al., 2018a), buccal swab samples (Li et al., 2016; van der Meulen et al., 2018a), oral washings (Alam et al., 2020; van der Meulen et al., 2018b; Zhou et al., 2018b), and tongue swab samples (de Paiva et al., 2016). The findings of these previous studies are diverse but indicate a lower abundance of *Haemophilus* (Alam et al., 2020; Li et al., 2016; van der Meulen et al., 2018a; Zhou et al., 2018b), *Neisseria* (Li et al. 2016; Rusthen et al., 2019; van der Meulen et al., 2018a; Zhou et al., 2018b), and *Lautropia* (Alam et al., 2020; van der Meulen et al., 2018a), and a higher abundance of *Veillonella* (Rusthen et al., 2019; Siddiqui et al., 2016; Singh et al., 2021; Zhou et al., 2018b) in patients with pSS compared to healthy controls. The results of a previous study also indicate that the spatial organization of microbial communities is shaped by the low whole saliva flow rates in Sjögren’s syndrome (Proctor et al., 2018).

In this study, we hypothesized that patients with pSS present a microbiome profile that is distinctively different from that of patients with non-pSS sicca and healthy controls across multiple oral sites, including stimulated whole saliva (SWS), supragingival plaque (SGP), buccal mucosa (BLM) and tongue scrape from the dorsal surface of the tongue (TGS), and this reflects the oral health status in terms of caries activity, gingivitis and oral infections. We investigated this by analyzing the oral microbiome samples by means of V3–V4 16S rRNA gene amplicon sequencing.

## Materials and Methods

### Study design and participants

This cross-sectional study included 59 female patients, who were all referred with suspicion of pSS by rheumatology and ophthalmology outpatient clinics or from dentists in private practice. Twenty-three patients fulfilled the American College of Rheumatology/European League against Rheumatism (ACR-EULAR) classification criteria for pSS (Shiboski et al., 2017), and 36 patients with Sjögren-like symptoms including oral and ocular dryness, did not fulfill the classification criteria for pSS (onwards designated non-pSS sicca). Additionally, we included 21 age- and gender-matched healthy control subjects. The inclusion criteria comprised age between 18 and 75 years for all participants, and no history of autoimmune diseases, xerostomia and hyposalivation and no intake of xerogenic medication for healthy controls. Exclusion criteria included use of antibiotics within the past 3 months, pregnant and nursing females, and patients with periodontal pocket depth of ≥ 6 mm on ≥ 4 teeth. The study was performed in accordance with the Declaration of Helsinki and approved by the Ethical Committees for the Region of Copenhagen, Denmark (H-18042385) and the Danish Data Protection Agency. General Data Protection Regulation (GDPR) was applied in the processing of personal data. Written informed consent was obtained from all participants.

### Clinical examination and sample collection

All participants were examined by one investigator (SK) at the Clinic for Oral Medicine, Department of Odontology, the Faculty of Health and Medical Sciences, University of Copenhagen, calibrated with and assisted by a senior investigator (AMLP). All participants underwent a detailed standardized interview regarding overall health, daily intake of medicine, and use of tobacco and alcohol. Questionnaires including Bother 1 index (BI), Bother index 5 (BI5) and Xerostomia Inventory (XI) were completed to assess the severity of oral dryness (xerostomia) (Challacombe et al., 2015; Osailan et al., 2012). The clinical examination included registration of periodontal pocket depth, gingival index and plaque index on six index teeth, as well as registration of decayed, missed, filled teeth (DMF-T) and -sites (DMF-S) (for details, Pedersen et al., 1999). Unstimulated whole saliva was collected for 15 min using the draining method and chewing-stimulated whole saliva was collected for 5 min (as previously described, Pedersen et al., 1999). The collected saliva samples were immediately kept on ice, then aliquoted and stored at -80°C until analysis. Tongue scrapes and supragingival plaque were collected and dissolved in Tris-EDTA (TE) buffer, then stored at -20°C until analysis. A biopsy (5 × 5 × 4 mm) was obtained from the buccal mucosa under local anesthesia and stored in TE-buffer at -20°C until further analysis.

All participants underwent a minor salivary gland biopsy in the lower lip for histopathological evaluation, including focus scoring (for details Daniels, 1984; Kamounah et al., 2024; Pedersen et al., 1999).

### DNA extraction and 16S rRNA gene amplicon sequencing

NucleoSpin Soil DNA kit (Macherey-Nagel, Germany) was used to extract genomic DNA from the bacterial pellets and negative controls. To lyse the bacterial cells in the pellet, the pellets were resuspended with optional enhancer SX solution and SL1 buffer, and lysed using Tissuelyser II (Qiagen, Germany) at a speed of 30 oscillations/sec for 5 min. The quality of extracted DNA was assessed using NanoDrop and Qubit Fluorometer (Thermo Fisher Scientific, USA). The V3–V4 variable region of 16S rRNA genes was sequenced using Illumina HiSeq 2500 (by BGI, China).

We recognize that 16S rRNA gene amplicon-based sequencing has its biases and pitfalls, as it may not accurately reflect absolute bacterial abundances, nor does it always possess the resolution for species/strain level resolution of certain taxa. Choice of variable regions covered by the amplicon influences these biases. In this study, we chose V3–V4 regions (longer amplicon but slight reduction in nucleotide accuracy in the middle of the amplicon region) over V4 region (shorter amplicon but high nucleotide accuracy across the entire amplicon region) because these longer amplicons provide improved species resolution for members of oral microbiome, e.g. *Streptococcus* spp. Sequencing near full-length V1–V8 amplicons using PacBio long-read technology will provide high accuracy as well as better taxonomic resolution, but it is prohibitively expensive, which precluded us from choosing that option.

### Sequence quality control

DADA2 (v1.24.0) (Callahan et al., 2016) was used with default parameters for initial processing of reads which consisted of paired Illumina HiSeq 2500 amplicon sequencing reads representing the V3–V4 region of the 16S rRNA genes. Primers were removed, and the reads were filtered and trimmed after assessing quality. A truncation length of 260 bases for the forward reads and 210 bases for the reverse reads was determined. The high-quality filtered and trimmed reads were then dereplicated and merged per sample after chimeras were identified and removed. We used the removeBiomeraDenovo() method from the DADA2 package in R to identify chimeric sequences and remove them. We use the consensus method, with parameters ‘minSampleFraction = 0.4, minFoldParentOverAbundance = 1.2’.

While supragingival plaque, buccal mucosa and tongue scrape microbiomes were sequenced in a single batch of Illumina HiSeq 2500 run, SWS samples were sequenced in a separate batch. Therefore, we had to estimate the sequencing error rates separately for SWS. This error rate is a mandatory input for the core sample inference algorithm of DADA2, which thus bifurcates the rest of the analysis. Thus, we generated ASVs for the two different batches separately. After the initial DADA2 processing, 38874 unique ASVs were identified in SWS samples, and 95229 ASVs from the other three sites. There were 15987 ASVs in common.

This process was followed by the removal of chimeric ASVs. After chimera removal, 2373 ASVs were identified in the saliva samples, and 6129 ASVs in the remaining samples. There were 1779 ASVs in common. At this stage, we proceeded to assign taxonomy to these ASVs.

### Taxonomic assignment

Taxonomy was assigned at the species level by means of the SILVA database (Quast et al., 2013), using ‘silva_nr99_v138.1_train_set.fa.gz’ for annotation at the genus level, and another database ‘silva_species_assignment_v138.1.fa.gz’ for species level resolution of taxonomic assignment.

We used the DADA2 Pipeline to taxonomically annotate ASVs using the SILVA database. We used the assignTaxonomy() function to annotate up to genus level, and then assignSpecies() function to add possible species annotation, both from the dada2 R package. The assignTaxonomy() function uses a naive Bayes RDP classifier (Wang et al. 2007) to classify reads into appropriate taxonomies, while the assignSpecies() function uses 100% sequence identity to annotate ASVs at the species level.

### Identification and filtering of ASVs

**Fig. 1.**
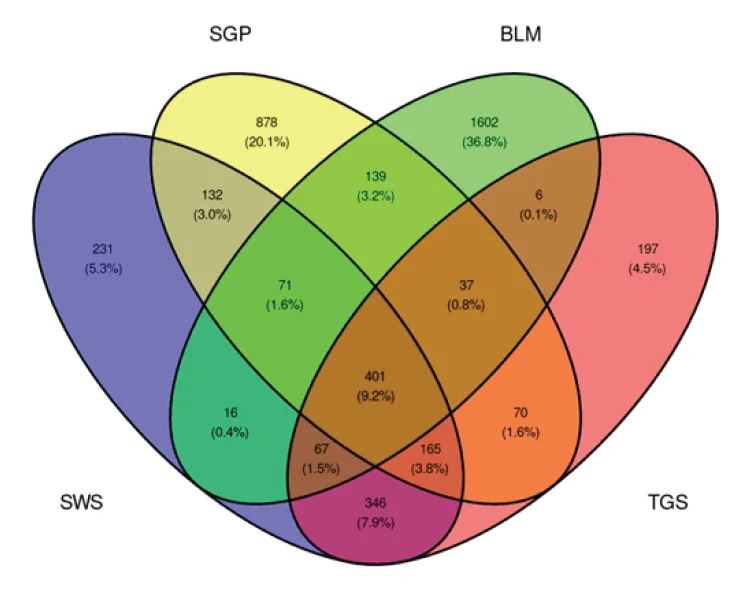
Venn Diagram showing the number of ASVs unique to each sampling site and shared ASVs across sites. ASV, amplicon sequence variant; SWS, stimulated whole saliva; SGP, supragingival plaque; BLM, buccal mucosa; TGS, tongue scrapes.

After taxonomic assignment, ASVs that were annotated as mitochondria and chloroplast were removed. Within each site, low abundant (< 0.0001 relative abundance) ASVs were removed. Additional filtration of ASVs according to their length resulted in 1429 ASVs for the SWS samples, 1893 for the SGP samples, 2339 for BLM samples, and 1289 for the TGS samples (Fig. 1).

### Alpha and beta diversity

The phyloseq (v1.40.0) package (McMurdie and Holmes, 2013) was used for easy manipulation of the data, and helper functions from this package were used to compute alpha diversity measures (estimate_richness()) and beta diversity (plot_ordination()). The vegan (v2.6-2) package (Oksanen et al., 2022) was also used to compute the Bray-Curtis dissimilarity between samples for beta diversity (vegdist()). The R library *microbiome* (v1.18.0) (Lahti and Shetty, 2022) was also used for calculating relative abundances from count data (abundances()).

### Graphing and image generation

Most images were generated in R using the packages ggplot2 (v3.3.6) (Wickham, 2016). The ggrepel (v0.9.1) (Slowikowski et al., 2021) package was used to ensure clean and neat labelling of plots with minimal overlap and clutter, and the ggpubr (v0.4.0) (Kassambara, 2020) package was used to additionally make plots cleaner, and combine multiple relevant plots in a single image.

### Statistical analysis

The *adonis2* function in the R vegan package (Oksanen et al., 2022) was used for PERMANOVA analysis. P-values were FDR-adjusted for multiple hypothesis testing. ANCOM from the package ANCOMBC was used (v1.6.4) to analyze the composition of the microbiome to obtain significantly different ASVs between conditions (that is pSS, non-pSS sicca, and healthy controls) while controlling for age as a confounder (Lin and Peddada, 2020). This statistical test accounts for variation included by age.

The clinical data from the pSS, non-pSS sicca and healthy control groups was compared by means of two-sample t-test (in case of normal distribution), and non-parametric Mann-Whitney U-test when data was not normally distributed. GraphPad Prism 10 was used as statistical software for these analyses.

We applied the widely used Benjamini-Hochberg method for controlling the false discovery rate (FDR) due to its balance between statistical power and error control, making it particularly suitable for high-dimensional datasets where multiple hypothesis testing is performed. FDR-adjusted P-value < 0.05 was considered statistically significant.

## Results

### Demographic and clinical characteristics of the participants

Table 1 summarizes the demographic, clinical, and pSS-related characteristics of the enrolled cohort. As 93% of the participants were females, with only two male participants in each group, we excluded male participants in statistical analysis to avoid reporting trends that may not be generalized due to this strong bias. At the time of diagnosis, the median age of the pSS cohort was 58 years. There were no significant differences between the patients with pSS and non-pSS in terms of age, gender, tobacco use, or severity of xerostomia. In addition, unstimulated and stimulated whole saliva flow rate values, the number of patients with hyposalivation, the score of gingival inflammation, and DMF-T/-S did not differ significantly between the two patient groups. The prevalence of keratoconjunctivitis sicca (p = 0.001), serum anti-SSA antibodies (p < 0.001), and labial salivary gland focus score ≥ 1 (p < 0.001) was significantly higher in patients with pSS than in patients with non-pSS sicca. The stimulated and unstimulated whole saliva flow rates were significantly lower in patients with both pSS (p < 0.001) and non-pSS sicca (p < 0.001) than in the healthy controls. DMF-T (p = 0.009) and DMF-S (p = 0.01) scores were significantly higher in the patients with pSS than in healthy controls, while no significant differences were found between pSS and non-pSS sicca nor non-pSS sicca and healthy controls.

### Measures of microbiome diversity within and across samples

**Fig. 2.**
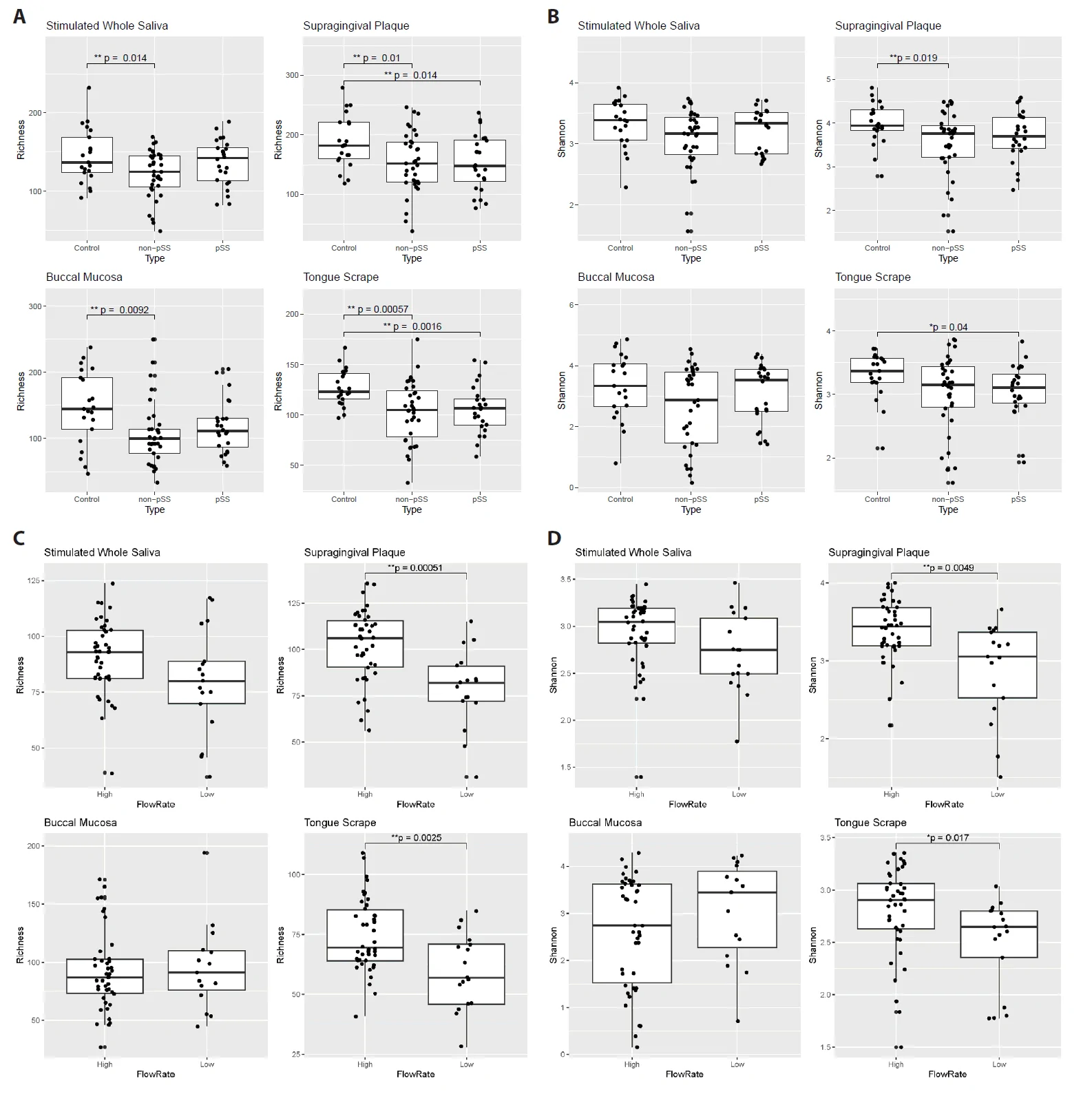
Comparison of microbiome alpha diversity measures across patient groups for the four sample sites. A, B ASV richness (A) and ASV Shannon diversity (B) between disease states (pSS, non-pSS and healthy controls). C, D ASV richness (C) and ASV Shannon diversity (D) between low- and high-flow rate subjects.

After processing the sequencing read data, alpha diversity was calculated for each of the four oral sites, and differences between groups of patients are shown in Fig. 2. Comparing pSS, non-pSS sicca and healthy controls, ASV richness was significantly lower in non-pSS sicca patients compared to healthy controls in all four sites (SWS, SGP, BLM, and TGS), with reduction in median richness of 12, 30, 45, and 18 ASVs, respectively (Fig. 2A). A corresponding reduction in Shannon diversity was only observed in SGP microbiomes (Fig. 2B). ASV richness was also lower in pSS patients compared to healthy controls in SGP and TGS microbiomes, with a reduction in median richness of 34 and 16 ASVs, respectively. A corresponding reduction in Shannon diversity was observed in TGS microbiomes.

To further investigate the effect of the salivary flow rate, we regrouped patients with pSS and non-pSS subjects based on their stimulated whole saliva flow rates and performed additional comparisons. Patients with abnormally low stimulated whole saliva secretion were determined to be those with a flow rate ≤ 0.70 ml/min, while the remaining were categorized as having stimulated whole saliva flow rates within the normal range. Patients with lower saliva flow rate exhibited a reduction in microbial alpha diversity: their SGP and TGS microbiomes showed a reduction in ASV richness (Fig. 2C, difference between median: 24 and 14, respectively) as well as Shannon diversity (Fig. 2D).

**Fig. 3.**
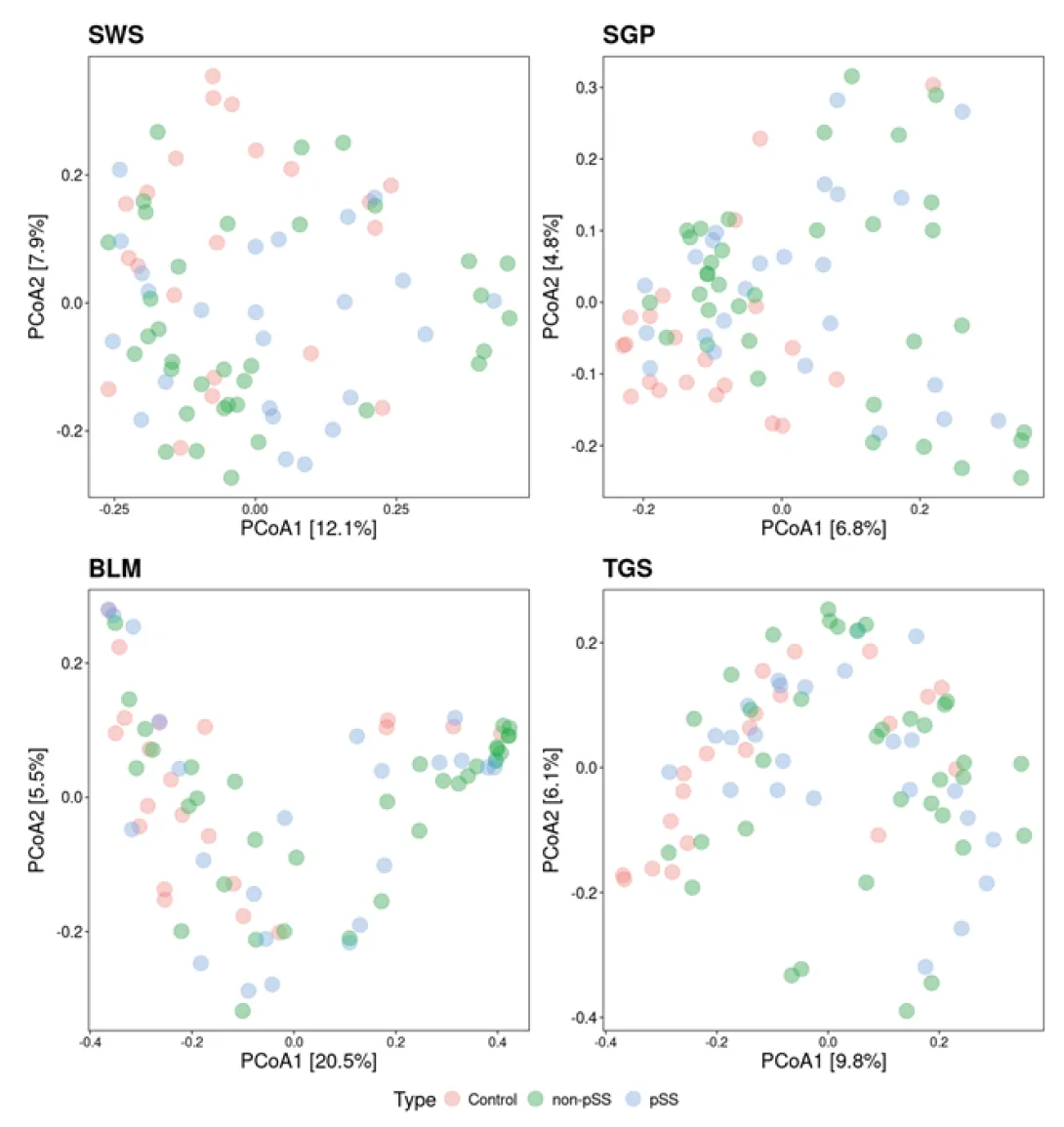
Beta diversity of samples across the different sampling sites and disease states (pSS = blue dots, non-pSS = green, and healthy controls = pink dots). SWS, stimulated whole saliva; SGP, supragingival plaque; BLM, buccal mucosa; TGS, tongue scrapes; pSS, primary Sjögren’s syndrome.

Beta diversity analysis was then performed with the Bray-Curtis dissimilarity being used as a measure of distance between samples (Fig. 3). To further investigate the effect of the diagnosis on beta diversity, PERMANOVA was performed, to investigate the amount of variation explained in the beta diversity by the disease state. In all four oral sites, the diagnosis had a significant effect on the beta diversity for SWS (p = 0.028), for SGP (p = 0.001), for BLM (p = 0.005), and for TGS (p = 0.007), explaining between 4.2% and 6.5% of the variance (Table 2). Similarly, saliva flow rate had a significant effect on the beta diversity for all four sites (p < 0.05), explaining between 2.4% and 3.4% of the variance (Table 3).

### Differentially abundant microbial features

**Fig. 4.**
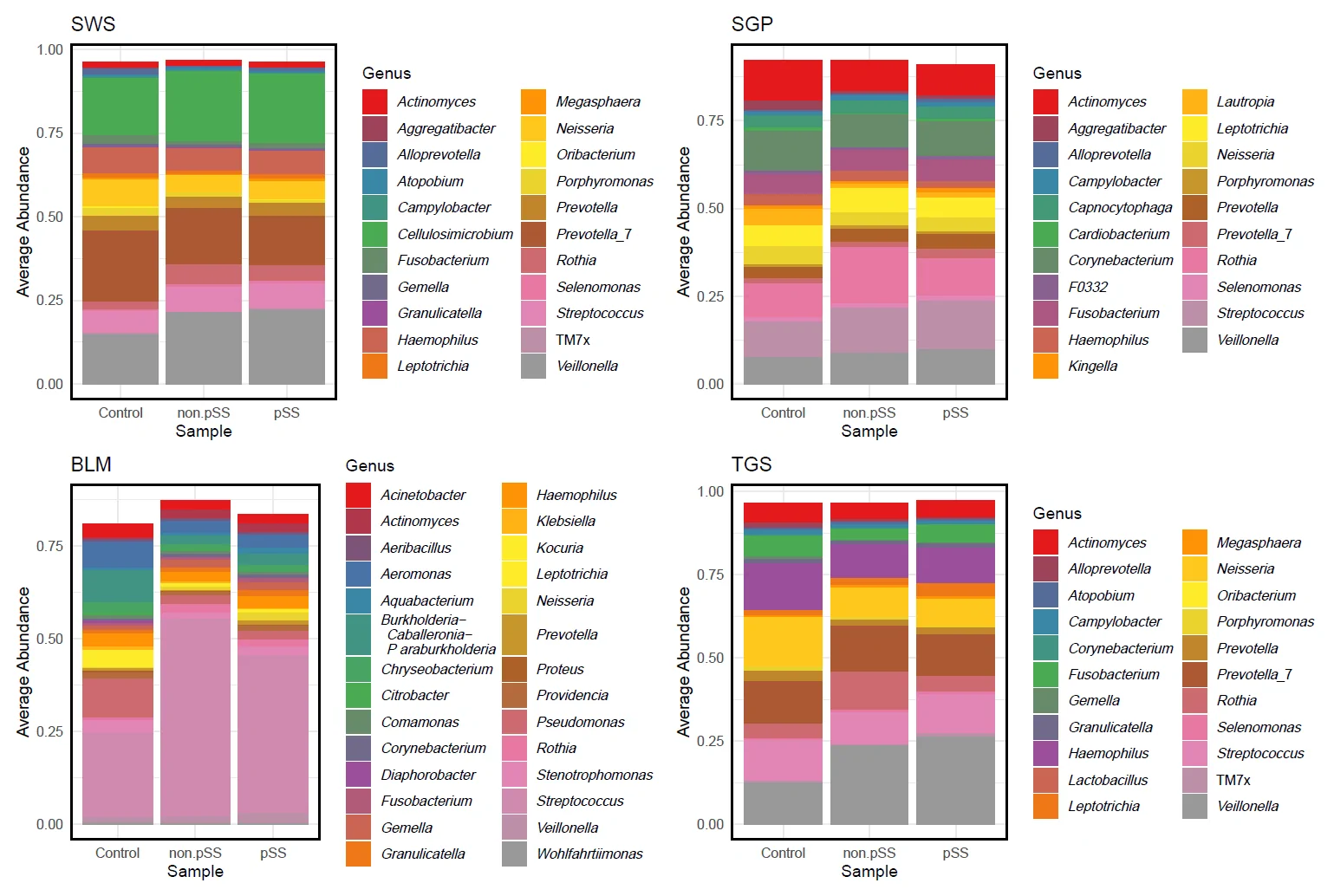
Group-wise mean genus relative abundance across different sites, with samples grouped by diagnosis. SWS, stimulated whole saliva; SGP, supragingival plaque; BLM, buccal mucosa; TGS, tongue scrapes.

**Fig. 5.**
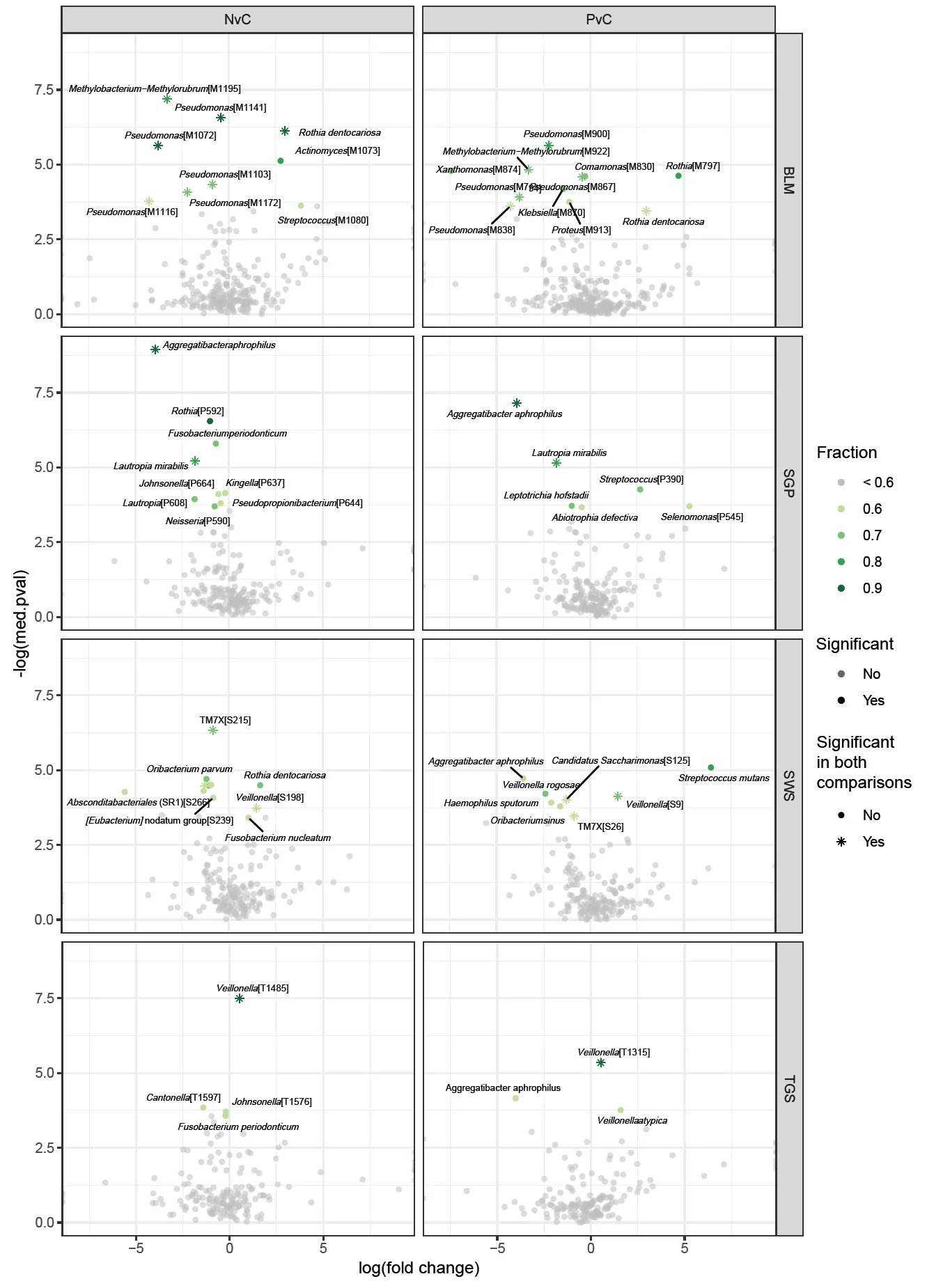
Differentially abundant ASVs from the ANCOM analysis comparing pSS or non-pSS samples with healthy controls in the four sample sites. log(fold change), log fold change of a given ASV between the two groups; -log(med.pval), the negative logarithm of the median p-value of the specific ASV; fraction, how often this ASV was significant in differential abundance tests when using every other ASV as the normalizing baseline (fraction > 0.6 is considered significant); pSS, primary Sjögren’s syndrome; NvC, non-pSS sicca versus healthy controls; PvC, pSS versus healthy controls; BLM, buccal mucosa; SGP, supragingival plaque; SWS, stimulated whole saliva; TGS, tongue scrapes.

The average genus-level composition of the groups showed that different genera dominated different sites (Fig. 4; see Fig. S1 for sample-level genus composition). While the average pSS and non-pSS compositions looked very similar, some genus level differences are apparent between pSS and control (or) non-pSS versus healthy controls. To investigate potential ASVs that are associated with diagnosis (pSS, non-pSS, and control), a differential abundance analysis was conducted for each oral site across the three groups. A total of 61 significantly different ASVs across sites was identified, including 28 ASVs differing significantly between patients with pSS and healthy control, and 33 ASVs between patients with non-pSS sicca and healthy controls (Table S1 and Fig. S2). Nearly half from this list of ASVs belonged to just five genera: *Pseudomonas*, *Veillonella*, *Rothia*, *Aggregatibacter*, and *Fusobacterium*. We found no significant differences in ASVs between pSS and non-pSS sicca. In the SWS samples, there were 8 significantly different ASVs between pSS and healthy controls, and 11 significantly different ASVs between non-pSS sicca and healthy controls. In the SGP samples, there were 6 significantly different ASVs between pSS and healthy controls, and 9 significantly different ASVs between non-pSS and healthy controls. In TGS samples, there were three significantly different ASVs between pSS and healthy controls, and four significantly different ASVs between non-pSS sicca and healthy controls. In BLM samples, there were 11 significantly different ASVs between pSS and healthy controls, and 9 significantly different ASVs between non-pSS sicca and healthy controls. The results of this test are summarized in the volcano plot (Fig. 5). The abundance of *Veillonella* species, including *V. atypica*, was significantly higher in SWS and TGS from patients with pSS and non-pSS sicca compared to healthy controls. In addition, the abundance of *V. rogosae* was lower in SWS from pSS patients compared to healthy controls.

To further investigate the effect of the salivary flow rate, we performed an additional differential abundance analysis between patients with lower and higher saliva flow rate. Patients with abnormally low stimulated whole saliva secretion were determined to be those with a flow rate ≤ 0.70 ml/min, while the remaining were categorized as having stimulated whole saliva flow rates within the normal range. Performing this analysis across the four sites revealed three significantly different ASVs in SWS, 11 in SGP, 8 in TGS, but none in BLM (Table 4 and Fig. S3).

## Discussion

In this study, we hypothesized that the microbiome is altered in multiple oral niches in patients with pSS. Overall, our data demonstrated many similarities but also some interesting differences compared to previous reported studies on the oral microbiome in patients with pSS. To our knowledge this is the first study to compare the microbiome in four different oral sites in patients with pSS compared to disease (non-pSS) and healthy control groups.

We found no significant differences between the two patient groups, pSS and non-pSS sicca, in terms of age, gender, tobacco use, dental status (DMF-T and DMF-S), saliva flow rates, and presence and severity of xerostomia. Additionally, there were no statistically significant differences in alpha diversity between patients with pSS and non-pSS sicca. However, the alpha diversity of the microbial community in SGP samples was significantly lower in the non-pSS sicca group than in the healthy control group. Furthermore, the alpha diversity was significantly lower in TGS samples in patients with pSS than in healthy controls. Our findings suggest that the distinct microbiome profile is associated with salivary gland hypofunction and not the pSS disease entity itself. This hypothesis was further confirmed when subgrouping pSS and non-pSS sicca patients according to their salivary flow rate rather than diagnosis, in which we found several ASVs with significantly different abundance.

A previous study reported that 8 individual bacterial taxa (including *Haemophilus*, *Neisseria*, and *Lautropia*) lost their association with pSS when the stimulated whole saliva flow rate was considered, suggesting that their relative abundances most likely are related to low salivary flow rates (van der Meulen et al., 2018a). However, additional taxa such as *Granulicatella* were still significantly associated with pSS when compared to the healthy control group, and when stimulated whole saliva flow rates were accounted for, suggesting a disease-specific association to pSS (van der Meulen et al., 2018a). Siddique et al. reported a higher abundance of *Streptococcus* and *Veillonella* species in patients with pSS, who had normal salivary secretion, suggesting that disturbances in the oral microbiota can occur independently of hyposalivation in pSS (Siddiqui et al., 2016).

In the present study, we did not find significant differences in ASVs between patients with pSS and non-pSS sicca, but the abundances of several bacteria were significantly different in patients with pSS and non-pSS sicca in comparison to healthy control subjects. In accordance with our findings, a recent study reported no differences in the oral microbiome between patients with pSS and non-pSS sicca using whole saliva samples to sequence the V1–V3 region (Sembler-Møller et al., 2019). Furthermore, we did not find significant differences in the whole saliva flow rates between patients with pSS and non-pSS sicca whereas both the unstimulated and stimulated whole saliva flow rates were significantly lower in pSS and non-pSS patients than in the healthy controls. These findings are in line with the findings reported by van der Meulen et al. (van der Meulen et al., 2018a). Thus, this suggest that it is not the underlying disease of pSS per se that causes changes in the oral microbiota, but more likely reduced salivary flow and the associated lower buffering capacity and salivary pH that causes a bacterial shift towards a higher proportion of aciduric and acidogenic lactic acid bacteria. This hypothesis was further supported by subgrouping patients based on their salivary flow rates (SWS > 0.7 ml/min versus SWS ≤ 0.7 ml/min), which revealed several significantly different ASVs between the two groups, in particular members of the lactic acid bacteria (Table 4). These findings are in accordance with those of a previous study suggesting that the low salivary flow rates in Sjögren’s syndrome shape the spatial gradient of bacterial diversity (Proctor et al., 2018).

A total of 61 ASVs in four oral sites differed significantly between healthy controls and patients with pSS and non-pSS sicca, including 19 ASVs in SWS, 15 in SGP, 20 in BLM and 7 in TGS samples. The levels of several *Veillonella* species, including *V. atypica*, were significantly higher in SWS and TGS in patients with pSS and non-pSS sicca than in healthy controls. These findings are in concordance with previous studies reporting a higher abundance of *Veillonella* in patients with pSS (Siddiqui et al., 2016; Singh et al., 2021; Xie et al., 2024; Zhou et al., 2018b). *Veillonella* species are Gram-negative bacteria anaerobic cocci characterized by their lactate-fermenting ability (Delwiche et al., 1985) and one of the most common genera in dental cavities. They are closely associated with acidogenic bacteria e.g., *Streptococci *(Aas et al., 2005; Belda-Ferre et al., 2012; Zhou et al., 2021). *Veillonella* species are known to have a bridging role in the oral biofilm and may facilitate the colonization of other pathogenic microorganisms in the oral cavity (Zhou et al., 2021). In our study, patients with pSS and non-pSS sicca had higher DMF-T/DMF-S scores than the healthy controls (although not significant for non-pSS sicca), reflecting a high caries experience, and therefore it is not surprising that the abundance of several *Veillonella* species were higher in the two patient groups. It further agrees with our observation that salivary levels of *V. rogosae* were lower among pSS patients compared to healthy controls, as this species is reportedly enriched in persons who are caries-free (Zhou et al., 2021). Our study also showed a reduction of *Haemophilus sputorum* in SWS microbiomes of pSS patients and reduction of *Neisseria* in SGP microbiomes of non-pSS patients, supporting the findings of previous studies (Li et al., 2016; van der Meulen et al., 2018a; Xie et al., 2024; Zhou et al., 2018b). It has been shown that patients with hyposalivation have a lower abundance of *Neisseria* compared to patients with normal salivary flow rates (Hayashi et al., 2014). *Neisseria* and *Haemophilus* exhibit low acid resistance and are not associated with caries. The relative abundance of both genera was found to be two-fold higher in patients with low caries experience than in patients with high caries experience (Belstrøm et al., 2017). *Rothia* species are predominantly observed in the oral cavity of healthy individuals and present in the initial biofilm (Zhou et al., 2018b). It has been shown that patients with pSS have a lower abundance of *Rothia* than healthy control subjects in oral rinse samples (Zhou et al., 2018b). Interestingly, we found a higher abundance of *Rothia dentocariosa* in SWS samples and BLM samples in patients with non-pSS sicca than in healthy controls, as well as a higher abundance of *Rothia dentocariosa* and *Rothia aeria* in BLM samples in patients with pSS than in healthy controls. However, the abundance of *Rothia species* in SGP samples was significantly lower in patients with non-pSS sicca than in healthy controls. A very recent study reported a higher relative abundance of *Leptotrichia* in saliva from patients with pSS than in healthy controls, and a higher relative abundance in plaque samples from patients with pSS, suggesting that *Leptotrichia* is related to dental caries in patients with pSS (Xie et al., 2024). However, we did not find a higher relative abundance of *Leptotrichia* on the genus level in any of the four sites, but a significantly lower relative abundance of *Leptotrichia hofstadii* in SGP samples in patients with pSS compared to healthy controls. Furthermore, we also found lower relative abundance of *Leptotrichia* in patients with abnormal low salivary secretion (SWS ≤ 0.7 ml/min) (Table 4).

*Streptococcus mutans* are Gram-positive cocci that are well-known for playing an important role in the formation of an anaerobic biofilm and are some of the primary pathogens of dental caries in humans (Hamada and Slade, 1980; Kawabata and Hamada, 1999). As expected, we found higher levels of *Streptococcus mutans* in SWS samples from patients with pSS, as these patients have a high caries experience. Furthermore, the patients with pSS had significantly lower whole saliva flow rates and higher DMF-T/DMF-S than in the healthy controls. In addition, our findings of a higher relative abundance of *S. mutans* in patients with pSS group agrees with the findings of previous studies (Almståhl et al., 2003; Kolavic et al., 1997). Interestingly, we found higher levels of *Streptococci *sp. on the dorsal surface of the tongue in patients with stimulated salivary flow rate > 0.7 ml/min than in those with flow rate ≤ 0.7 ml/min (Table 4).

Furthermore, we also found significantly higher levels of Lactobacillus in SWS, SGP, and TGS samples in patients with a SWS rate ≤ 0.70 ml/min, which is consistent with previously published literature that has also reported higher levels of Lactobacillus sp. in patients with low salivary flow rate (Almståhl et al., 2003; Guobis et al., 2011; Kolavic et al., 1997; Lundström and Lindström, 1995) (Table 4).

Overall, our study showed that there are no statistically significant differences in ASVs between patients with pSS and non-pSS sicca, but significant differences in multiple bacteria in all four sites between both patient groups and healthy controls, and in three sites (SWS, SGP, and TGS) between patients with high (> 0.7 ml/min) versus low (≤ 0.7 ml/min) stimulated whole saliva flow rates. Additionally, we identified several ASVs with significant different abundance when regrouping patients according to their salivary flow rate. Thus, our findings suggest that severe salivary gland hypofunction rather than pSS itself, modifies the composition of the oral microbiota in site-specific patterns. This can also explain why the two patient groups (pSS and non-pSS) were similar with respect to presence of oral diseases/conditions (caries, gingivitis etc.).

## Figures and Tables

**Table 1.** Demographic and clinical characteristics of the patients with pSS and non-pSS sicca and healthy control (HC) subjects. Values are given in mean ± SD, median (range) and in numbers of patients (%)

	pSS (n =23)	non-pSS (n =36)	Healthy controls (n=21)	P-value
Age (years)	58 ± 14	57 ± 12	58 ± 14	N.S.
Female	23 (100%)	36 (100%)	21 (100%)	
Current smokers	6 (26%)	11 (31%)	1 (5%)	N.S.*, **
				0.02***
Dry eyes for > 3 months	21 (91%)	31 (86%)	0 (0%)	N.S.*
				<0.001**, ***
Dry mouth for > 3 months	19 (83%)	31 (86%)	0 (0%)	N.S.*
				<0.001**, ***
Xerostomia Inventory score	39 (16–53)	40 (16–55)	13 (11–18)	N.S.*
				<0.001**, ***
Hyposalivaton (UWS ≤ 0.10 ml/min)	9 (39%)	13 (36%)	0 (0%)	N.S.*
				0.002**
				0.005***
Keratoconjunctivitis sicca	20 (87%)	10 (28%)	N.A.	0.001*
					Positive serum anti-SSA/Ro antibody,	20 (87%)	9 (25%)	N.A.	<0.001*
Focus score ≥ 1	16 (70%)	0 (0%)	0 (0%)	<0.001*, **					
				N.S.***
UWS (ml/min)	0.18 ± 0.16	0.20 ± 0.18	0.46 ± 0.30	N.S.*
Median (range)	0.18 (0.005–0.63)	0.18 (0.01–0.94)	0.36 (0.12–1.19)	<0.001**, ***
SWS (ml/min)	1.04 ± 0.73	1.46 ± 1.00	2.21 ± 0.79	N.S.*
Median (range)	0.82 (0.08–2.92)	1.29 (0.16–4.62)	2.26 (1.1–3.9)	<0.001**, ***
Plaque index	0.72 ± 0.55	0.42 ± 0.24	0.34 ± 0.14	0.03*,
Median (range)	0.5 (0.04–2.13)	0.33 (0.04–1.13)	0.33 (0.08–0.63)	0.005**,
				N.S.***
Gingival index	0.74 ± 0.66	0.50 ± 0.34	0.41 ± 0.24	N.S.
Median (range)	0.58 (0–2.67)	0.5 (0–1.29)	0.29 (0.13–0.88)	
DMFT	17.09 ± 7.64	14.42 ± 7.29	11.24 ± 5.55	N.S.*, ***
Median (range)	18 (2–28)	13 (0–28)	12 (0–21)	0.009**
DMFS	56.44 ± 38.43	42.56 ± 32.87	30.33 ± 20.92	N.S.*, ***
Median (range)	53 (7–138)	30 (0–140)	26 (0–63)	0.01**

Comparison analyses *pSS vs non-pSS, ** pSS vs HC** and ***non-pSS vs HC. DMFT, Decayed, Missing, Filled Teeth; DMFS, Decayed, Missing, Filled Surfaces; SD, standard deviation; UWS, unstimulated whole saliva flow rate; SWS, chewing-stimulated whole saliva flow rate.

**Table 2.** Proportion of variance in beta diversity explained by the disease state (pSS and non-pSS) in all four sites using the PERMANOVA analysis

Sample site	R^2^	P-adj
Stimulated whole saliva	0.04209	0.028
Supragingival plaque	0.04846	0.001
Buccal mucosa	0.06539	0.005
Dorsal surface of the tongue	0.04288	0.007

R^2^, variance explained; P-adj, FDR-adjusted p-value for the F statistics.

**Table 3.** Proportion of variance in beta diversity explained by the salivary flow rate in all four sites using the PERMANOVA

Sample site	R^2^	P-adj
Stimulated whole saliva	0.02986	0.005
Supragingival plaque	0.02608	0.002
Buccal mucosa	0.02371	0.049
Dorsal surface of the tongue	0.03363	0.002

R^2^, variance explained; P-adj, FDR-adjusted p-value for the F statistics.

**Table 4.** List of ASVs significantly associated with salivary flow rate ≤ 0.70 ml/min. ASVs are annotated at genus level (and when possible at species level)

Stimulated whole saliva	Supragingival plaque	Dorsal surface of the tongue
· *Fusobacterium periodonticum *	· *Leptotrichia* [P30]	· *Fusobacterium* *periodonticum*
· TM7x (Family Saccharimonadaceae) [S31]	· *Corynebacterium* [P31]	· *Leptotrichia* [T30]
· *Lactobacillus* [S163]	· *Ligilactobacillus* [P150]	· *Streptococcus* [T45]
	· *Lactobacillus* [P168]	· *Ligilactobacillus* [T153]
	· *Scardovia* *wiggsiae*	· *Lactobacillus* [T171]
	· *Limosilactobacillus* [P200]	· *Scardovia* *wiggsiae*
	· *Atopobium* *parvulum*	· *Limosilactobacillus* [T201]
	· *Lactobacillus* [P335]	· *Lacticaseibacillus* [T429]
	· *Parascardovia* *denticolens*	
	· *Lacticaseibacillus* [P398]	
	· HT002 (Family Lactobacillaceae) [P399]	

ASVs marked with red were significantly more abundant in patients with salivary flow rate ≤ 0.70 ml/min. ASVs marked with blue were significantly less abundant in patients with salivary flow rate ≤ 0.70 ml/min.

## Data Availability

The data supporting the findings of this study is provided in the manuscript and the supplementary table. The 16S rRNA gene amplicon sequence files have been deposited at NCBI Short Read Archive under BioProject identifier PRJNA1176379.

## Supplementary Information

Fig. S1. Heatmap of relative abundances of top 20 genera per site, grouped by disease state, and showing all individual samples.

Fig. S2. (A) Heatmap of relative abundances of significantly different ASVs per site, between pSS and healthy controls, grouped by disease state. (B) Heatmap of relative abundances of significantly different ASVs per site, between non-pSS and healthy controls, grouped by disease state.

Fig. S3. Heatmap of relative abundances of significantly different ASVs per site, between patients with high and low flow rates.

Table S1. Glycans detected after B-elimination of stimulated saliva samples from three controls and three patients. Peeling products(p) and N-glycans (n) have been excluded

Table S2. MSAC metrics of saliva from patients and healthy controls
