# Supplementary material for: Microbial signatures in oral sites of patients with primary Sjögren’s syndrome: Association with salivary gland hypofunction
Source: J Microbiol. 2025 Jun 30;63(6):e2501030. doi: 10.71150/jm.2501030 (PMC13577787; doi:10.71150/jm.2501030)

Supplementary Figures

Fig. S1. Heatmap of relative abundances of top 20 genera per site, grouped by disease state, and showing all individual samples.

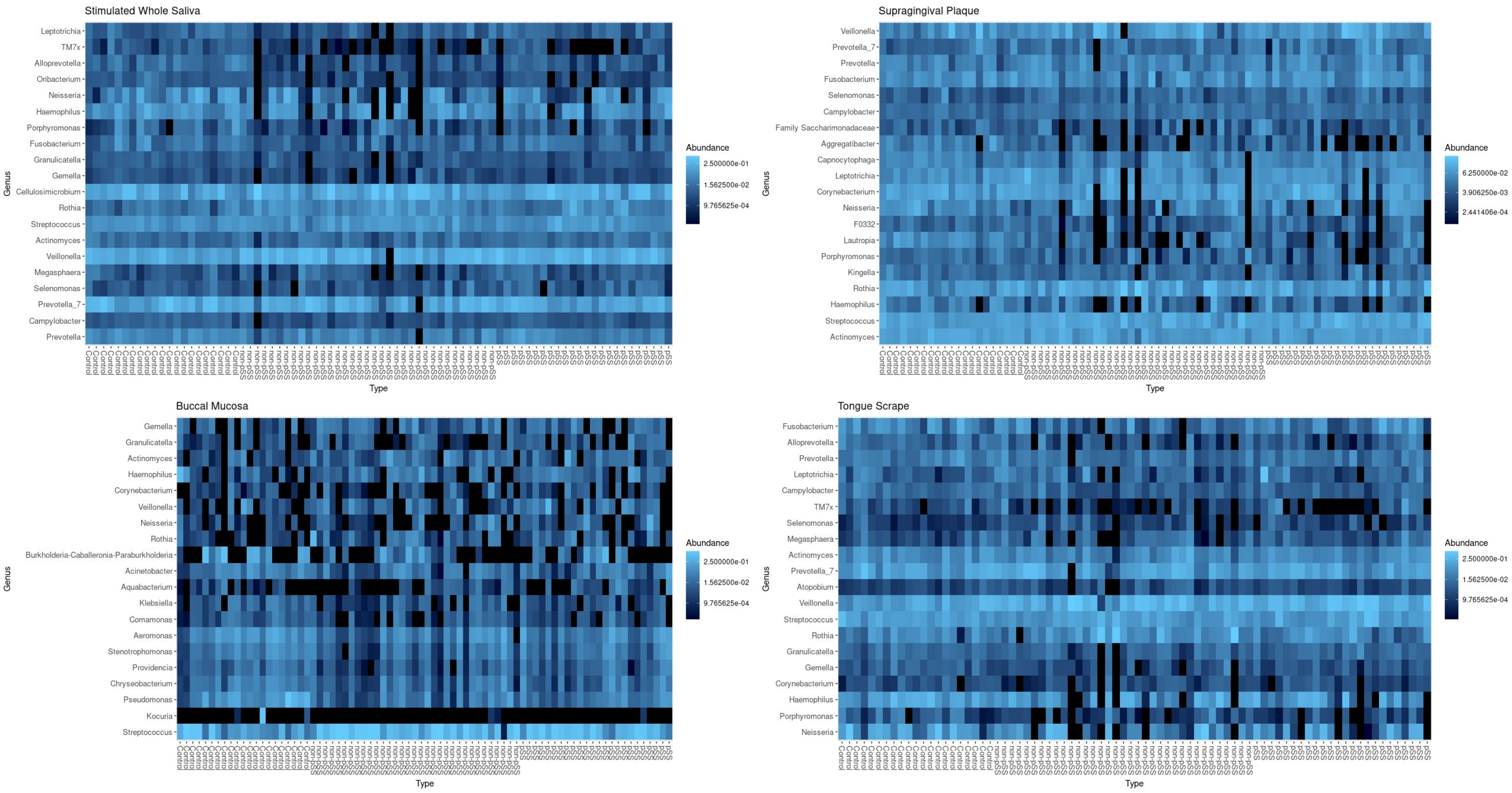

Supplement: Supplementary file 1 [file jm-2501030-Supplementary-Fig-S1.pdf]
