# Supplementary material for: Microbial signatures in oral sites of patients with primary Sjögren’s syndrome: Association with salivary gland hypofunction
Source: J Microbiol. 2025 Jun 30;63(6):e2501030. doi: 10.71150/jm.2501030 (PMC13577787; doi:10.71150/jm.2501030)

Fig. S2.

(A) Heatmap of relative abundances of significantly different ASVs per site, between pSS and healthy controls, grouped by disease state.

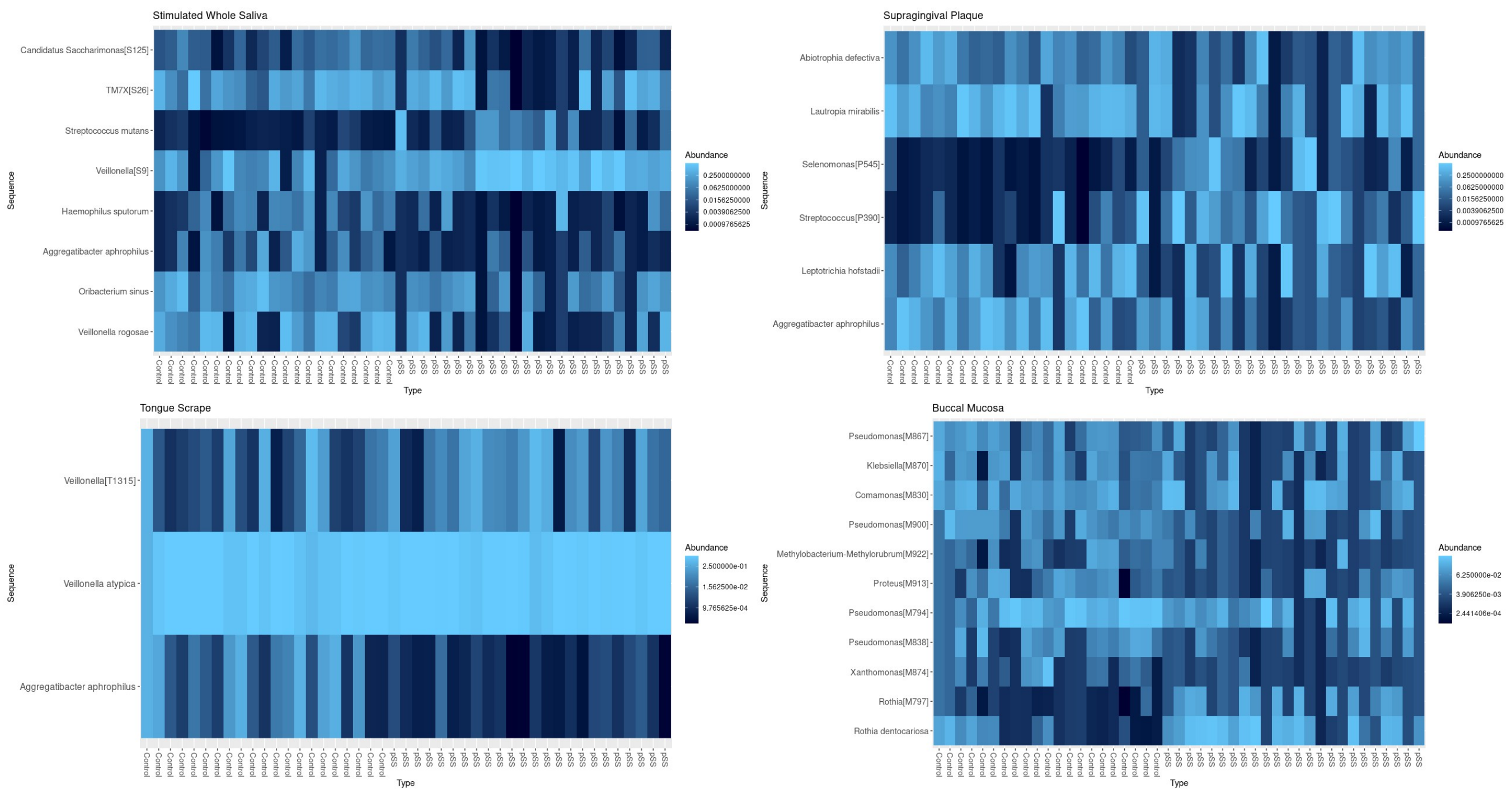

(B) Heatmap of relative abundances of significantly different ASVs per site, between non-pSS and healthy controls, grouped by disease state.

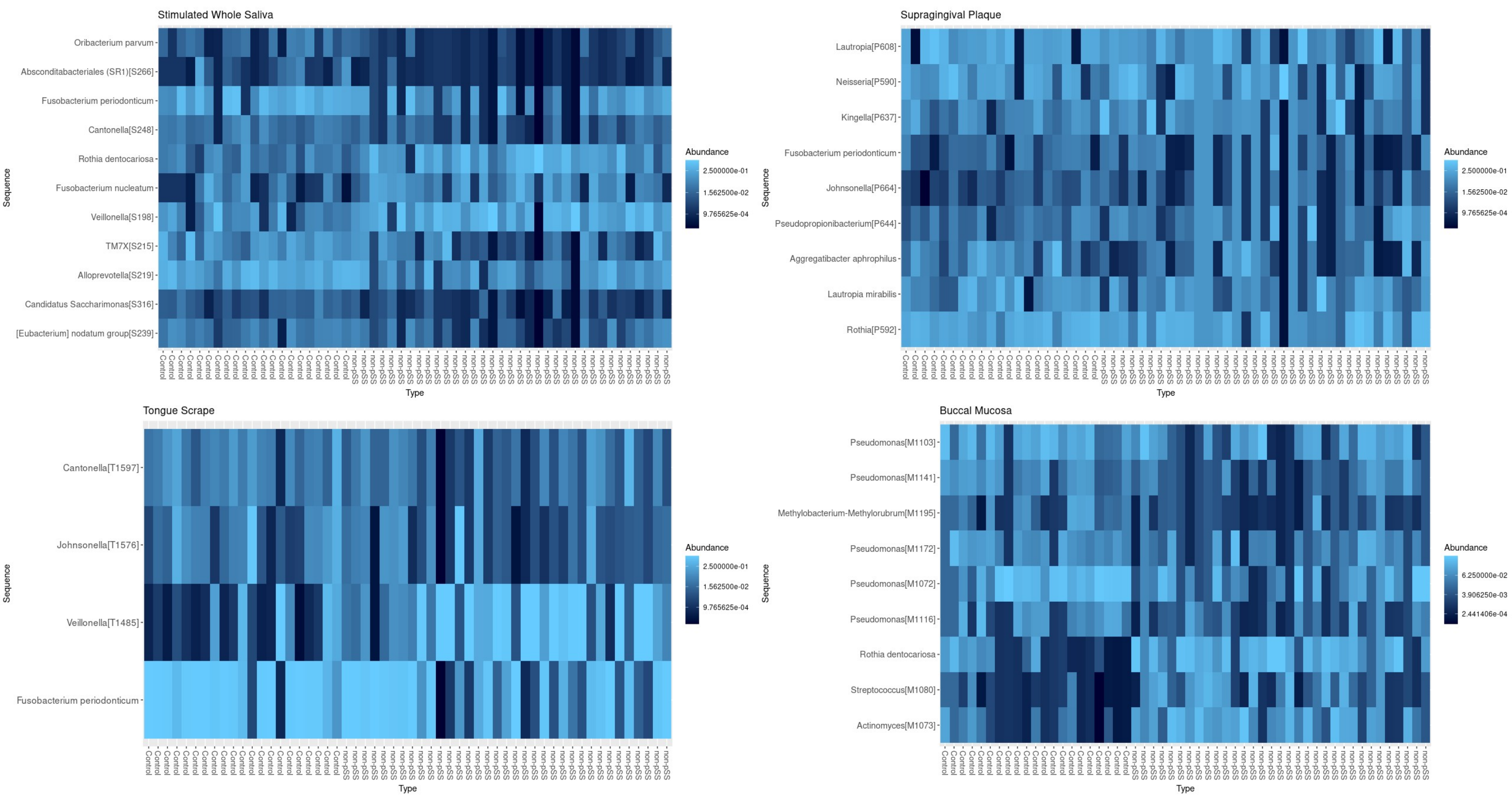

Supplement: Supplementary file 2 [file jm-2501030-Supplementary-Fig-S2.pdf]
