# Supplementary material for: Microbial signatures in oral sites of patients with primary Sjögren’s syndrome: Association with salivary gland hypofunction
Source: J Microbiol. 2025 Jun 30;63(6):e2501030. doi: 10.71150/jm.2501030 (PMC13577787; doi:10.71150/jm.2501030)

Fig. S3. Heatmap of relative abundances of significantly different ASVs per site, between patients with high and low flow rates.

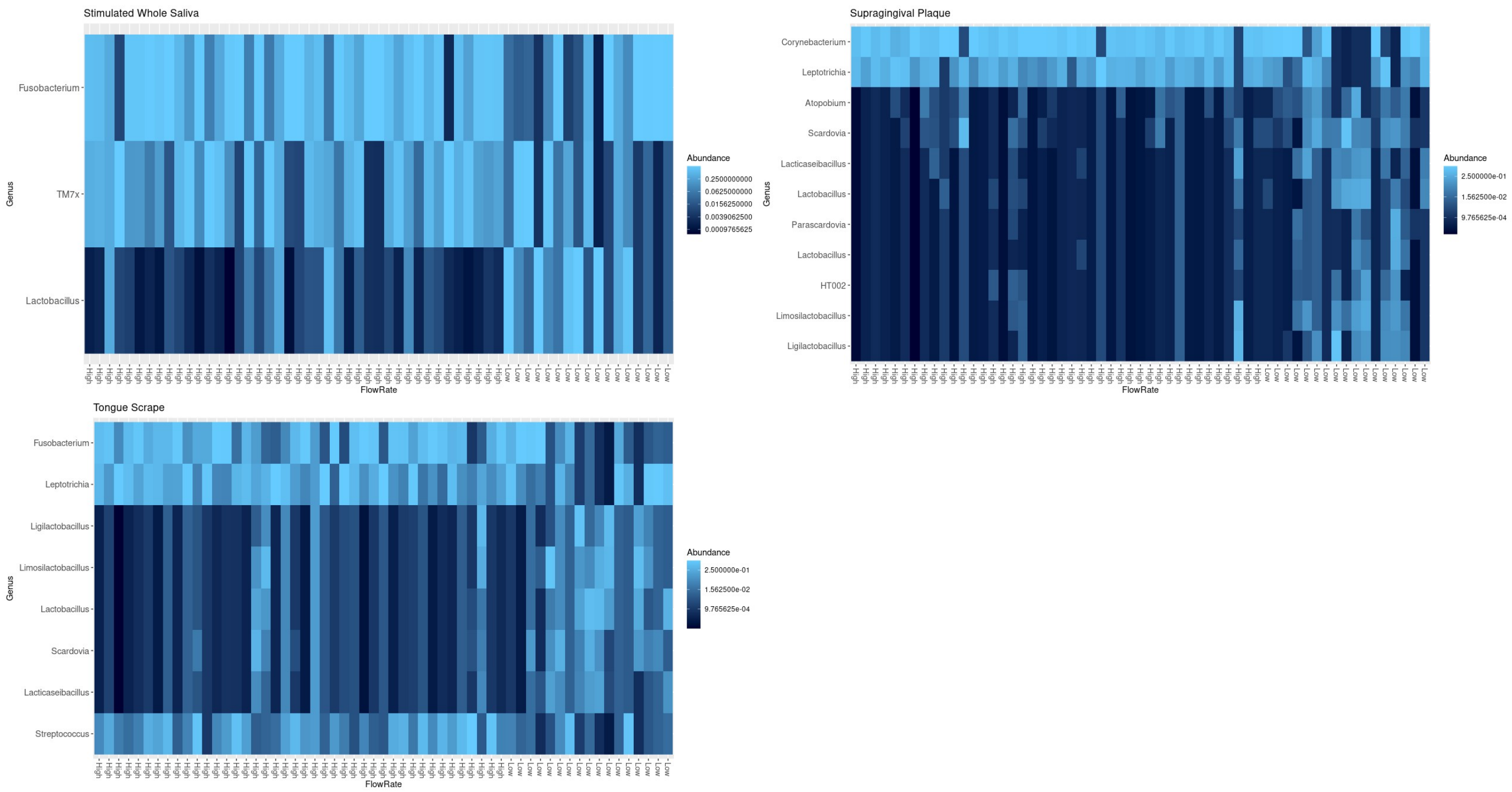

Supplement: Supplementary file 3 [file jm-2501030-Supplementary-Fig-S3.pdf]
